# Neural activity during free association to conflict–related sentences

**DOI:** 10.3389/fnhum.2013.00705

**Published:** 2013-10-25

**Authors:** Aram Kehyayan, Katrin Best, Jo-Birger Schmeing, Nikolai Axmacher, Henrik Kessler

**Affiliations:** ^1^Department of Epileptology, University of BonnBonn, Germany; ^2^Department of Psychosomatic Medicine and Psychotherapy, University of BonnBonn, Germany; ^3^German Center for Neurodegenerative DiseasesBonn, Germany; ^4^Department of Psychosomatic Medicine and Psychotherapy, LWL University Hospital, Ruhr-University BochumBochum, Germany

**Keywords:** neuropsychoanalysis, psychoanalysis, neuroscience, free association, fMRI, operationalized psychodynamic diagnostics, emotion processing

## Abstract

Psychodynamic conflicts form an important construct to understand the genesis and maintenance of mental disorders. Conflict-related themes should therefore provoke strong reactions on the behavioral, physiological, and neural level. We confronted *N* = 18 healthy subjects with a vast array of sentences describing typical psychodynamic conflict themes in the fMRI scanner and let them associate spontaneously in reaction. The overt associations were then analyzed according to psychoanalytic theory and the system of operationalized psychodynamic diagnosis and used as a genuinely psychodynamic indicator, whether each potentially conflict-related sentence actually touched a conflict theme of the individual. Behavioral, physiological, and neural reactions were compared between those subjects with an “apparent conflict” and those with “absent conflicts.” The first group reported stronger agreement with the conflict-related sentences, more negative valence in reaction, had higher levels of skin conductance reactivity and exhibited stronger activation in the anterior cingulate cortex, amongst other functions involved in emotion processing and conflict-monitoring. In conjunction, we interpret this activity as a possible correlate of subjects’ inherent reactions and regulatory processes evoked by conflict themes. This study makes a point for the fruitfulness of the neuropsychoanalytic endeavor by using free association, the classical technique most commonly used in psychoanalysis, to investigate aspects of conflict processing in neuroimaging.

## INTRODUCTION

This study is a contribution in the field of neuropsychoanalysis. The term itself was coined in 1999 with the inauguration of the respective journal and can broadly be defined as the attempt to bridge psychoanalysis and neuroscience on multiple layers. Pioneers in the field started with investigations of patients with focal brain damage with techniques derived from psychoanalysis (Kaplan-Solms and Solms, 2000). Later on, studies were concerned with the investigation of phenomena described by psychoanalysis (e.g., repression, dreams) with neuroscientific methods (Solms and Turnbull, 2011). Many books and reviews have been written that cover theoretical as well as empirical aspects of this new scientific field (Solms and Turnbull, 2002; Fotopoulou et al., 2012; Panksepp and Solms, 2012). This is a broad new field with studies ranging from psychoanalytic observations in patients with brain lesions (Kaplan-Solms and Solms, 2000) to investigations on the neural changes during psychoanalysis (Buchheim et al., 2012). A comprehensive review of the empirical work in neuropsychoanalysis is provided by Sauvagnat et al. (2010). This work includes operationalizations of specific psychoanalytic concepts such as, e.g., dreams (Ruby, 2011), repression (Erdelyi, 2006), and primary-process thinking (Carhart-Harris and Friston, 2010).

In this study, we focus on an approach that has been termed “psychoanalytically informed neuroscience” – i.e., the testing of concepts associated with psychoanalysis with neuroscientific methods (Solms and Turnbull, 2011). This study investigates the concept of “psychodynamic conflict” using the (originally therapeutic) method of free association to potentially conflict-related contents inside the fMRI scanner. Being a major component of psychoanalytic theory and practice, psychodynamic conflicts represent a powerful construct that helps understanding mental disorders, their genesis and eventual “maintenance” (Person et al., 2005). Different biographical experiences may lead to the formation of psychodynamic conflicts, which consist of a main theme (e.g., the tension between the desire for autonomy and the need for dependency, or difficulties to adequately value one’s self-esteem). Such conflicts comprise of typical core affects, modes of transference and counter-transference, and span wide areas of the subject’s life (family, friends, job, recreational, etc.). Hence, if a theme comprised in the subject’s conflict is touched in a real-life situation, reactions on the behavioral, cognitive, and physiological level should be expected, that call for the regulation of cognitions, impulses and, most importantly, emotions. Such regulatory processes can show varying degrees of consciousness depending on the conflict and subjects’ conscious coping with it (Brenner, 1982; Mentzos, 1984). The regulatory coping with conflict-related themes is well-understood in clinical contexts (Person et al., 2005) and guides many psychodynamic forms of psychotherapy (Strupp and Binder, 1985; Wöller and Kruse, 2010). The neural correlates of such processes have not been studied, though. Therefore, in this pilot study, we searched for neural and psychophysiological activation when subjects have to deal with conflict-related themes. In an economic first approach, we presented healthy subjects with a wide array of sentences depicting multiple potentially conflict-related themes and used their spontaneous associations to them as a genuinely psychodynamic indicator of whether the sentences actually touched a conflict-theme or not. Behavioral, psychophysiological, and neural reactions in only those subjects where an actual conflict is touched could then be a correlate of their subjective reactions to the conflict-related content or any regulatory processes mentioned above. In detail, we exposed healthy individuals sequentially to three different types of sentences in the MRI scanner and let them associate spontaneously what came into their minds after reading each sentence. The three conditions were neutral, unspecific negative emotional, and sentences that were also negative emotional but constructed in a psychoanalytically informed way to reflect possible psychodynamic conflicts. Part of the data obtained in this study has already been published (Schmeing et al., 2013) and showed that conflict-related sentences in general (i.e., irrespective of actual relevance for the subjects), among other effects, led to higher skin conductance reactivity (SCR) and enhanced activation of the anterior cingulate cortex/pre-supplementary motor area (ACC/pre-SMA) in the whole group of healthy subjects. Both effects could well be interpreted as being a correlate of subjects’ affective reactions and emotional processing. To increase specificity of results, though, the individual impact a given stimulus has on the subject has to be taken into account (Kessler et al., 2011a,b). As conflict-related sentences were constructed on a theoretical basis (psychoanalysis) reflecting common themes of autonomy/dependency and self-esteem, they were the same for all subjects. To disentangle the actual impact of the sentences, expert psychotherapists trained in psychoanalysis assessed the audio-taped free association to the conflict-related sentences of subjects post-hoc, based on their clinical experience and descriptions of manifestations of common psychodynamic conflicts as provided by the Operationalized Psychodynamic Diagnostics Manual (OPD-Task-Force, 2008). The guiding questions were: does this sentence actually touch a conflict theme apparent in the subject? Is it of relevance because the subject struggles with this theme, whether consciously or unconsciously? This analysis led to a separation of tested subjects into two groups: “apparent conflict” comprised individuals with at least one of the associations reflecting a possible psychodynamic conflict; “absent conflict” consisted of individuals who did not show signs of conflict in any of their associations. Details of the rating process can be found in the Section “Materials and Methods.” Derived from psychoanalytic theory and the results from our previous paper (Schmeing et al., 2013), we put forward the following hypotheses. Subjects in the “apparent conflict” group should exhibit stronger reactions on multiple levels (behavioral, physiological, brain activity) to the conflict-related sentences than those in the “absent conflict” group. One question regarding the role of psychodynamic conflicts for an individual will additionally be tested in this study: is “having” a conflict better described as a trait or a state? That is, will subjects in the “apparent conflict” group exhibit the hypothesized stronger activity in reaction to *all* conflict-related sentences, regardless of the specific association to that sentence, because they generally tend to react strongly to that sort of content (trait)? More specific, subjects in the “apparent conflict” group could have the strong reactions only to the sentences that have actually produced the salient associations (state). To test this, analyses were carried out in a between-subject design (comparing “apparent conflict” and “absent conflict” subjects) and a within-subject design (comparing “conflict” and “no conflict” sentences only within the “apparent conflict” group). In detail, we assumed that the “apparent conflict” group should evaluate the sentences with a more negative valence (behavioral). On the physiological level, skin conductance reflecting autonomic arousal should be relatively higher in “apparent conflict” subjects when confronted with conflict-related sentences. Finally, brain activity in the ACC/pre-SMA [amongst other functions relevant for emotion processing (Etkin et al., 2011) and conflict monitoring (Botvinick, 2007)] should be enhanced in “apparent conflict” subjects. Since the ACC/pre-SMA was relatively more active when associating to conflict-related sentences in the whole group of subjects (Schmeing et al., 2013), differential activity of this region for only the “apparent conflict” group would strengthen the specificity of our findings. To this end, this study represents, in essence, an analysis of the behavioral, physiological, and brain data obtained in Schmeing et al. (2013), guided by the separation of all subjects into two groups based on the psychoanalytic content of their free associations. Although the current study was based on the same dataset, it focuses on the analysis of the *content* of the participants’ free associations following conflict sentences, which was not investigated in our previous paper.

## MATERIALS AND METHODS

### ETHICS STATEMENT

The study was approved by the local medical ethics committee (“Ethikkommission an der Medizinischen Fakultaet der Rheinischen Friedrich-Wilhelms-Universitaet Bonn”), was according to the latest version of the Declaration of Helsinki, and all subjects provided written informed consent.

In **Box 1**, we provide an example of a free association from one participant, which was slightly modified to ensure that s/he cannot be identified. This participant signed an agreement that this text can be published.

**Box 1. An example of the clinical evaluation of trials.**

The following assessment of associations was performed based on the manual of operationalized psychodynamic diagnosis (OPD-Task-Force, 2008). The subject in the example shown here was classified as belonging to the “apparent conflict” group, consisting of individuals with at least one association reflecting a possible psychodynamic conflict. The following answer to one conflict sentence (“I only feel good when someone is actually taking care of me”) has been chosen to provide the reader with an understanding of the rating process used.

OPD-conflict: desire for care vs. autarchy, *passive mode*

Following the OPD manual (OPD-Task-Force, 2008, p. 227–230) subjects who suffer from desire for care vs. autarchy conflict in passive mode can be described as being “dependent and demanding.” They have the strong desire to be taken care of by others. As can be seen in the following example, such subjects tend to show high emotional attachment to others in their lives, try to avoid being alone or feeling lonely. In relationships, they can experience difficulties when separated from their partners. If the partner sets boundaries on the limits of care he/she is willing to provide, this can lead to feelings of depression, insufficiency, and fear of being alone.

Free associations of subject to the stimulus sentence: “I only feel good when someone is taking care of me:”

“Loneliness ⋯ to be left alone ⋯ social attachment. This sentence applies to me fully. *I believe I am a person who finds it difficult to be alone* (author comment: subject starts speaking more quietly) ⋯ I am a person who prefers being surrounded by people that aren’t my first choice, rather than being alone. This was one of the issues that came up in my relationships. Both with my current girlfriend as well as with my previous girlfriend – we often had the issue that in the evenings she ⋯ well ⋯ let’s say ⋯ leaves me by myself in front of the TV and goes to bed ⋯ or when she withdraws into normal domestic life and I am looking for much more contact ⋯ *well I am not the type of person with whom one can be in the same room for 2–3 hours and not talk,* *like someone who just sits in a corner* ⋯ (author comment: subjects is getting upset, speaking more loudly)”

### PARTICIPANTS

Participants were recruited through notifications on the homepage of the University of Bonn Students’ Service. They were paid 10€/h (total time for the experiment 3.5–4 h). They were right-handed, native German speakers with normal or corrected-to-normal vision, and without current or past neurological or psychiatric disorders.

In the experiment, a total of 23 subjects were scanned, 5 of which were excluded from analysis because of high motion artifacts (more than one voxel diameter; 3 subjects), or early interruption of the experiment (2 subjects). Of the 18 participants included in both fMRI and behavioral/SCR analysis (10 female), mean age was 25.9 ± 3.2 years (mean ± standard deviation).

### Experimental Paradigm

All participants of the study were invited a few days before the experiment in order to practice the technique of free association and to screen them for psychiatric symptoms. For this screening, two questionnaires were used: SCL-90 (symptom check list) and BDI (Beck’s Depression Inventory). Those who scored high on either of the questionnaires (cut-offs: BDI >11, SCL-90 Global Severity Index >0.57) were excluded from the experiment.

The experiment consisted of three parts: association phase (including a rating), break/distraction, and memory recall.

#### Association phase

Subjects were placed in the MRI scanner, with video goggles to present stimuli (Nordic Neuro Lab, Bergen, Norway), a microphone to record verbal response (Fibersound^®^ Microphone Model FOM1-MR and Fibersound^®^ Control Model FOM1-DRx Battery/wall powered; Micro Optics Technologies Fibersound^TM^ Audio, Middleton, WI, USA), and two electrodes connected to the right palm for SCR measurements. A hand-held four-button device was used for rating. A stimulus (one of 24 sentences, presented in random order) was shown for 5 s, followed by a 60 s time period (indicated by a question mark) for free association. During this total period of 65 s, the verbal responses of the subjects were digitally recorded. The participants were asked to say the first three words that came to their mind after stimulus presentation, and use the remaining time for (overt) free association. Afterwards, subjects rated their agreement with the sentence (on a scale ranging from 1: very strong disagreement to 9: very strong agreement), and their own emotional state after association in terms of valence (-4: very negative to +4: very positive feeling) and arousal (1: very calm to 9: very aroused). Rating was followed by a 30-s break. After an inter-stimulus-interval (fixation cross) of 1.5–3 s, the next stimulus was presented.

Of the 24 stimulus sentences, 6 were “neutral” and 6 were “generally negative,” while the remaining 12 were “conflict related,” meaning that they were designed to resemble typical expressions of psychodynamic conflicts. Those conflicts were selected on the basis of psychoanalytic theory and specified using the system of operationalized psychodynamic diagnosis (OPD; Cierpka et al., 2007; OPD-Task-Force, 2008). OPD is an instrument for the assessment of psychodynamic constructs (e.g., relation, conflict, structure). Two types of conflict were used for the generation of conflict-related sentences: autonomy/dependency (e.g., “I cannot say ‘No’ if someone else is asking me for help”), and self-esteem–conflict (e.g., “I often estimate myself as little competent”). Those two conflicts have been selected since they are most common among subjects suffering from interpersonal problems (Cierpka et al., 2007) and are operationalized in a very stringent and comprehensive way in the OPD manual. For each conflict, the manual provides anchor examples of typical manifestations regarding partnership, family, profession, behavior in groups, and others. The anchor examples served as a basis for the formulation of our stimulus sentences. Additionally, a state-licensed psychoanalyst not otherwise involved in the study confirmed the relevance of our stimuli. The neutral sentences described situations of mildly positive to neutral emotional content (e.g., “I try to follow the news on a regular basis”). The “generally negative” sentences included situations with negative value that could not typically be associated with a psychodynamic conflict (e.g., “Sometimes I am frightened when I walk alone in the dark”). A list of all sentences is provided as **Table 1**.

**Table 1:** List of sentences.

Neutral sentences
Occasionally I like to watch movies on the television
I try to follow the news on a regular basis
Sometimes my mood is influenced by the weather
There are topics I am more interested in than politics or economy
Mostly I do respect the traffic regulations
I find it important to find the time for my hobbies once in a while
**Negative sentences**
I am getting annoyed when I am stuck in a traffic jam and I have an important appointment
Sometimes I am frightened when I walk alone in the dark
When an overtaking car on the other side of the street approaches me, my heart sinks into my boots
Sometimes I become sad, when I think about dead soldiers in the war
Seeing a helpless animal suffer often makes me sad
When somebody is pushing in the line, it can really upset me
**Conflict sentences: desire for care vs. autarchy (passive)**
All my life I got a raw deal
I wish that finally someone is taking care of me
I have the feeling that I always get too little
I actually only feel good when someone is taking care of me
**Conflict sentences: desire for care vs. autarchy (active)**
I give so much, without getting really rewarded
I cannot say “No” if someone else is asking me for help
I do not need anything or anybody to be happy
I hate it to be a burden for other people
**Conflict sentences: self-value**
Usually I have a very low self esteem
I am often embarrassed about myself
Sometimes I am disgusted by myself
I often estimate myself as little competent

#### Break/distraction

During the 1-h break/distraction phase outside the MRI scanner, subjects filled out the DSQ-40 questionnaire, designed to assess the prominence of maladaptive, adaptive, and neurotic defense mechanisms. There was no difference in DSQ-40 scores between the “apparent conflict” and “absent conflict” groups. This phase was mainly designed to distract subjects before the upcoming unexpected memory recall task.

#### Memory recall

After the break, subjects had to perform an unexpected memory recall task. Again, they were placed in the MRI scanner with video goggles, microphone, and SCR-electrodes. All 24 sentences were presented again, after each of which subjects had 30 s to remember and name the 3 words that had come to their mind to that sentence in the beginning of the association phase in the first part of the experiment (not the content of the following free association phase). Again, answers were recorded via microphone. Only the first three answers were evaluated, and participants were encouraged to guess if they were unsure. The memory task was included because one of our original hypotheses, investigated in Schmeing et al. (2013), had been that associations with a long reaction time, and accompanied by a high SCR, were less likely to be remembered afterwards (see also Levinger and Clark, 1961; Rossmann, 1984; Kohler and Wilke, 1999). This subsequent forgetting may be a marker of repression during the free association period. In the current manuscript, activity related to cues was assessed regardless of the success of subsequent memory recall, because there was no difference in memory either between the “apparent conflict” and “absent conflict” groups or between “apparent conflict” and “absent conflict” trials within the first group.

Subjects were rewarded with 0.10€ for each correct answer afterwards, and for each incorrect or missing answer 0.05€ were subtracted from their total gain. Since audio recordings of association and recall had to be compared individually (by listening to them) in order to check if memory recall was correct, subjects received no immediate feedback about their performance.

Trials were considered valid and included into analysis if the participants had given three associations at the beginning of the association phase, and audio quality was good enough in both association phase and recall phase to allow for comparison. Successful memory recall was not required for trials to be considered valid and to be included into analysis.

### MRI DATA ACQUISITION AND ANALYSIS

Thirty-four axial slices were collected at 1.5 T (Avanto, Siemens, Erlangen, Germany). We collected T2^*^-weighted, gradient echo EPI scans (slice thickness: 3.0 mm; voxel size: 3 mm × 3 mm × 3 mm; matrix size: 64 × 64; field of view: 210 mm × 210 mm; repetition time: 2700 ms; echo time: 40 ms). Thereafter, we acquired a 3D-sagittal T1-weighted MPRAGE sequence for each subject for anatomical localization (number of slices: 160; slice thickness: 1 mm; inter-slice gap: 0.5 mm; voxel size: 1 mm × 1 mm × 1 mm; matrix size 256 × 256; field of view: 256 mm; echo time: 3.09 ms; repetition time: 1660 ms).

Activity was analyzed during the free association phase of the experiment, but not during the recall phase.

MRIs were pre-processed in SPM5 () using standard pre-processing steps including realignment, unwarping, normalization, and smoothing with a 6-mm Gaussian kernel. Pre-processed data were fitted by the convolution of multiple regressors with a canonical hemodynamic response function to obtain parameter estimates for each condition covariate.

Two different GLMs were used to compare reactions to conflict sentences between the “apparent conflict” and “absent conflict” group (conflict as a trait) and to compare reactions to conflict sentences that actually triggered a conflict and those that did not trigger a conflict within the “apparent conflict” group (conflict as a state).

Separate regressors were used to model transient activity directly after cue presentation (delta pulses, i.e., stick functions with a duration of *t* = 0, triggered to the onset of cue presentation) and more sustained activity related to free association (box-car regressors ranging from 5 s after stimulus onset until the end of a trial) for the neutral, negative, and conflict conditions. To model early activation, we used stick functions instead of box-car regressors in order to maximize comparability with our previous analyses reported in Schmeing et al. (2013), where stick functions were used as well. The rationale for this analysis is that we expected repression effects to occur rapidly after stimulus presentation, because cues trigger internal conflicts rapidly and before a participant has finished generating a word for free association. This is supported by our findings from Schmeing et al. (2013), showing that reaction times differ as a function of sentence category. The content of the subsequent free association (which was analyzed in the current study) may then be a marker of the actual conflict generated by each sentence. Since the box-car regressors for sustained activity did not reveal any differences between the conditions, all contrasts and results reported in the results section refer to the early, delta-pulse regressors.

For the comparison between the “apparent conflict” and the “absent conflict” group, we used the following set of regressors:

(1) Regressor triggered to the onset of conflict sentence presentation regardless of apparent conflicts; duration = 0
(2) Regressor triggered to the onset of negative sentence presentation; duration = 0
(3) Regressor triggered to the onset of neutral sentence presentation; duration = 0
(4) Regressor triggered to 5 s after the onset of conflict sentence presentation for free associations, regardless of apparent conflicts; duration = 60 s.
(5) Regressor triggered to 5 s after the onset of negative sentence presentation; duration = 60 s
(6) Regressor triggered to 5 s after the onset of neutral sentence presentation; duration = 60 s
(7) Regressor triggered to the onset of the rating periods after each free association period (i.e., triggered to 65 s after the onset of each sentence presentation); since rating was self-paced, the duration of this regressor was variable
(8) Regressor triggered to the onset of the inter-stimulus break; duration = 30 s.

Another GLM was calculated for the analysis within the “apparent conflict” group. Here, regressors 1 and 4 were split into “apparent conflict” and “absent conflict,” according to clinical evaluation. These are henceforth referred to as regressors 1A/1B and 4A/4B, respectively. All other regressors remained the same.

In our contrast analyses, we conducted two complementary approaches:

(A) Within the subgroup of participants who showed an apparent conflict in reaction to at least one conflict sentence, we contrasted beta values from regressor 1A vs. regressor 1B, and from regressor 4A vs. regressor 4B intra-individually. Then, a one-sample *t*-test was used to determine whether contrast means differed significantly from 0.
(B) For our between-groups analysis, we first calculated intra-individual contrasts between sentence conditions (regressor 1 vs. regressor 2; regressor 1 vs. regressor 3; regressor 2 vs. regressor 3; 2^*^regressor 1 vs. regressor 2 + regressor 3). Corresponding analyses were conducted for regressors 4–6. These contrasts were then compared between the “apparent conflict” and the “absent conflict” group, using a two-sample *t*-test.

**FIGURE 1:**
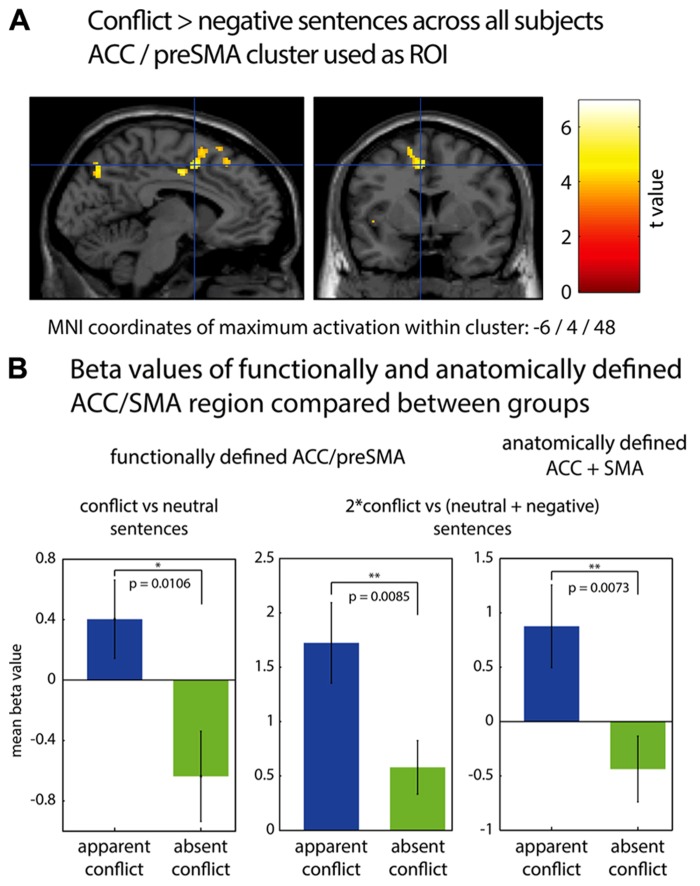
**(A)** fMRI cluster including parts of ACC and preSMA, found to be activated during association to conflict-related sentences compared to negative sentences (Schmeing et al., 2013). **(B)** Comparison of mean beta values in functionally and anatomically defined regions of interest between group 1 (“apparent conflict”) and group 2 (“absent conflict”), using a one-tailed two-sample *t*-test. Bar plots indicate mean values with SEM. ^*^p < 0.05; ^**^p < 0.01.

In **Figure 1**, fMRI results are displayed using neurological convention (left hemisphere on the left side of the figure). To identify significant activations, we used an uncorrected voxel threshold of *P* < 0.001 and an additional cluster threshold of *p* < 0.05, corrected for multiple comparisons using the false discovery rate (FDR) procedure of SPM5. For the ROI analyses, two sources to identify the ACC/pre-SMA were used. First, the ACC/pre-SMA area found to be activated by conflict sentences as compared to negative sentences for all subjects [MNI coordinates: -6/4/48; reported in Schmeing et al. (2013)]. It was chosen because one of our goals was to validate the findings of Schmeing et al. (2013), and to show that the ACC activation observed was indeed due to the personal relevance of conflict sentences to the subjects. Therefore, we had the hypothesis that this area should be more strongly activated in the “apparent conflict” group, for whom at least some of the conflict sentences were of personal relevance, according to our clinical rating. Second, an anatomically defined ROI of ACC/pre-SMA was applied according to the automatic anatomic labeling toolbox for SPM [AAL; Tzourio-Mazoyer et al. (2002)].

### SCR ACQUISITION AND ANALYSIS

We collected the SCR data with a sampling rate of 1000 Hz with BrainVision Recorder Software. Data were corrected for MRI-artifacts using BrainVision Analyser 2.0. We down-sampled data to 200 Hz and low-pass filtered them at 5 Hz. The corrected data were analyzed using the software LEDALAB (Benedek and Kaernbach, 2010) to extract phasic electrodermal activity in an integral of 4.5–13.1 s after stimulus presentation. This interval corresponds to the mean peak time of SCR-curves ± 0.5SD (8.8 ± 4.3 s).

### CLINICAL EVALUATION OF TRIALS

The recorded free-association periods of all 12 conflict-related stimulus sentences were assessed post-hoc by two expert psychotherapists trained in psychoanalysis, unaware of subjects’ ratings of agreement, valence, and arousal, and not involved in fMRI analysis (Katrin Best, Henrik Kessler). Assessment of associations was performed according to the manual of OPD (OPD-Task-Force, 2008) and building on psychoanalytic theory. The OPD manual provides detailed anchor examples of typical manifestations for each psychodynamic conflict, by listing emotions, thoughts, beliefs, behavior, transference, countertransference, relationship themes, and others that are most commonly associated with that conflict. Subjects’ associations were compared with the anchor examples for the respective conflict covered in the stimulus sentence. If, for example, the stimulus sentence is covering the “autonomy-dependency” conflict, then associations dealing with feelings of envy towards others that presumably received more than the subject would point towards that very conflict playing a role. Of course, in the reality of assessing associations things were more complex and considered various aspects of the material provided by the subjects (e.g., prosody, wording, breaks, hesitation). The aim was to detect associations that point to probable psychodynamic conflicts regarding the theme of the stimulus sentence. Obviously, those conflicts could be conscious or unconscious, hence leaving the opportunity for direct confirmation of the agreement, valence, or arousal in reaction to a given stimulus sentence via subjects’ ratings. The analysis of associations led to a separation of tested subjects into two groups: “apparent conflict” comprised individuals with at least one association reflecting a possible psychodynamic conflict; “absent conflict” consisted of individuals who did not show any sign of conflict in their associations. Trials with conflict-related sentences were only included into analysis if a decision could be reached whether the trial was to be classified as “apparent conflict” or “absent conflict.” Each association was evaluated completely by one expert (Katrin Best), and each rating was discussed with the second expert (Henrik Kessler). Whenever Katrin Best considered a rating insecure, it was independently evaluated by Henrik Kessler. Thus, all possible conflicts were thoroughly discussed between both experts until a consensus was reached. Because of our method of rating it is not possible to provide inter-rater reliability. An example of the evaluation process is provided in **Box 1**.

### STATISTICAL ANALYSIS

We conducted one-tailed tests if we had directed *a priori* expectations and two-tailed tests otherwise. In detail, we had the following directed hypotheses:

(1) In a between-group analysis, group 1 subjects (showing at least one “apparent conflict”) should in general react to conflict-related sentences with higher agreement, more negative valence, higher SCRs (autonomic arousal), and increased BOLD response in ACC/pre-SMA as compared to group 2 subjects (without any “apparent conflict” sentences).
(2) Within group 1, higher agreement, more negative valence, higher SCRs, and increased BOLD response in ACC/pre-SMA to sentences rated “apparent conflict” compared to sentences rated “absent conflict” should be evident.

## RESULTS

### BEHAVIORAL AND SCR

Based on our clinical evaluation, 23 out of 209 valid trials were rated as “apparent conflict,” equivalent to 11% of all trials. A proportion of 89% of all trials (186) were rated as “absent conflict.” 8 of our 18 subjects showed free associations classified as “apparent conflict” in at least one trial. Their number of “apparent conflict”-trials ranged from 1 to 7 (8.3–58.3% of their trials; mean: 2.88 trials; SD: 2.36 trials). In this subpopulation of subjects, 23 out of 89 trials (or 26%) were classified as “apparent conflict,” 66 were rated as “absent conflict” (74%).

At first, we looked for differences in behavioral measures between group 1 (those subjects who had an “apparent conflict” in at least one association period according to the clinical evaluation) and group 2 (those who showed no “apparent conflict” in their associations), comparing all trials with conflict-related sentences between those two groups. **Table 2** provides the results of this comparison. Subjects in group 1 reported a more negative mood after their association, indicated by more negative ratings of valence (*p* = 0.007, Mann–Whitney *U*-test, one-tailed), and showed a higher degree of agreement with the sentences compared with group 2 (*p* = 0.028, Mann–Whitney *U*-test, one-tailed). Also, they had significantly higher SCRs (*p* < 10^-^^4^, *t*_209_ = 4.15, two-sample *t*-test, one-tailed).

**Table 2:** Comparison of valence and agreement ratings and skin conductance reactivity between group 1 with “apparent conflict” and group 2 with “absent conflict.”

		Between-group analysis	
	Group 1 “apparent conflict”		Group 2 “absent conflict”		*p*	*t*
	Mean	SD	Mean	SD
Valence rating	-0.04	1.78	0.51	1.52	0.007	
Agreement rating	3.7	2.39	3.31	2.54	0.028	
SCR (μS^*^s)	14.8	14.3	8.3	8.4	<10^-^^4^	*t*_209_ = 4.15

*Valence and agreement compared using a Mann–Whitney *U*-test, SCRs compared using a two-sample *t*-test, both one-tailed.
*

Next, we investigated whether trials within group 1 subjects, classified by our clinical evaluation as “apparent conflict” or “absent conflict,” could be discriminated by behavioral measures. **Table 3** provides the results from this within-group analysis. Consistent with our findings from the between-groups analysis, valence ratings for “apparent conflict”-trials were more negative compared to “absent conflict”-trials (*p* < 10^-^^4^, Mann–Whitney *U*-test, one-tailed) and there was a higher agreement with “apparent conflict” sentences (*p* = 0.0033, Mann–Whitney *U*-test, one-tailed). Contrary to our hypothesis though, a difference in SCR was not observed.

**Table 3:** Comparison of valence and agreement ratings and skin conductance reactivity only within group 1 with “apparent conflict” between trials with and without “apparent conflicts.”

		Within-group analysis “apparent conflict” group	
	Apparent conflict trials		Absent conflict trials		*p*	*t*
	Mean	SD	Mean	SD
Valence rating	-1.39	1.59	0.38	1.60	<10^-^^4^	
Agreement rating	4.83	2.15	3.36	2.38	0.0033	
SCR (μS*s)	14.3	13.0	14.8	15.0	0.56	*t*_87_ = 0.16

*Valence and agreement compared using a Mann–Whitney *U*-test, SCRs compared using a two-sample *t*-test, both one-tailed.*

### FUNCTIONAL MRI RESULTS

We first performed a region of interest analysis of the ACC/pre-SMA area found to be activated by conflict sentences as compared to negative sentences [MNI coordinates: -6/4/48; reported in Schmeing et al., 2013]. **Figure 1** shows the location of this area and bar graphs depicting the comparisons. We found that in group 1 (with “apparent conflict”), this cluster was more strongly activated for conflict-related sentences compared to neutral sentences than in group 2 (*t*_16_ = 2.56; *p* = 0.0106; two-sample *t*-test, one-tailed) and for conflict-related sentences compared to both neutral and negative sentences (contrast: 2^*^conflict vs. [neutral + negative]; *t*_16_ = 2.66; *p* = 0.0085; two-sample *t*-test, one-tailed). An anatomical region of interest composed of bilateral ACC and SMA showed a difference between the groups for conflict-related sentences vs. both negative and neutral sentences (*t*_16_ = 2.74, *p* = 0.0073; two-sample *t*-test, one-tailed). Within-group comparisons between sentences comprising an “apparent conflict” and those with “absent conflict” yielded no significant results on the fMRI level. Also, there were no significant differences on the whole brain level either between the two groups or within group 1, with correction for multiple comparisons using the false discovery rate (FDR) procedure of SPM5.

## DISCUSSION

We presented a pilot study tapping into the concept of psychodynamic conflicts, in which we investigated the behavioral, physiological, and brain activation profiles during free association to putatively conflict-related sentences. All subjects were confronted with stimulus sentences describing typical psychodynamic conflict themes and had to associate spontaneously to them. Subjects’ associations were then analyzed based on psychoanalytic theory and the system of OPD (OPD-Task-Force, 2008). This led to their separation into two groups: Group 1 consisted of individuals where the conflict-related sentences actually touched a “sore spot,” meaning that the conflict is of relevance for them. Group 2 included subjects where associations to the sentences were free of any sign of psychodynamic conflict. With this individualized and psychoanalytically informed approach in data analysis, our results show differences between groups that can be interpreted as a possible correlate of psychodynamic conflict processing. In comparison to group 2, group 1 reported more agreement with the material of the conflict-related sentences, more negative valence and exhibited higher SCR. Additionally, subjects in group 1 specifically had enhanced activation in ACC/pre-SMA during processing of conflict sentences.

According to our hypothesis, specific activity in the “apparent conflict” group should reflect their reactions to the confrontation with psychodynamic conflicts and probably regulatory processes involved spontaneously. In clinical practice, the degree of consciousness of such reactions depends on the conflict itself and the level of coping with it (Brenner, 1982; Mentzos, 1984; Person et al., 2005). Since our subjects reported higher agreement with the contents covered in the conflict-related sentences, it is likely that their reactions and probable regulation take place consciously and that they are aware of the problems mentioned. It is therefore unlikely that our sentences touched unconscious (i.e., deeply buried, repressed) conflicts but rather the level of conflict where an awareness and ways to cope with it exist. Since we deliberately included only subjects without current or past psychiatric disorders, this result is not surprising, though. Yet, as “apparent conflict” subjects reported relatively more negative valence after the conflict-related sentences and their associations, the material presented had an emotional meaning and caused a subjective impact. The enhanced skin conductance reactions strengthen this effect and indicate that the confrontation with psychodynamic conflicts could have led to autonomic arousal. The latter point has already been hypothesized and in fact empirically shown by other researchers including Jung (Jung, 1918; Levinger and Clark, 1961; Kohler and Wilke, 1999). As for differences in BOLD responses, specific activity in the ACC for the “apparent conflict” group can also be interpreted in the vein of relatively conscious processing of conflicts. The ACC region with differential activity is, amongst other functions, involved in emotional processing in general (Murphy et al., 2003; Etkin et al., 2011) and is supposed to play a key role when attending to subjective emotional responses (Lane et al., 1997). Following the old dichotomy of ACC subdivisions (Bush et al., 2000), the area of our ACC activation lies in the cognitive subdivision. Recent conceptualisations, though, argue for the involvement of the whole ACC in emotional processing with the dorsal-caudal regions (where our area can be localized) reflecting appraisal of negative emotions (Etkin et al., 2011). This ACC activity could thus be a neural correlate of subjects’ emotional arousal in accordance with reports in self assessments (valence) and measured by skin conductance. In vein of this, our activation site lies in the dorsal anterior cingulate cortex (dACC), which also forms part of the so-called salience network (Seeley et al., 2007). Activity in this salience network might reflect subjects’ arousal when confronted with the conflict (salient) sentences. It might also be that enhanced dACC activation is part of a defensive process (repression), where subjects “block” true self-reflection. This speculation would be in line with the ideas expressed in Axmacher et al. (2010), where it is argued that repression hinders the integration of memories with self-referential processes. Additionally, the dACC has recently been discussed to be part of a brain system processing social disconnection and painful affects (neural alarm system concerning threat-related responding) in human relations (Eisenberger and Cole, 2012). Hence, the conflict-related sentences could have served as stimuli evoking memories or fears of social disconnection in some subjects. This line of interpretation would be well consistent with the psychodynamic interpretation that some conflict sentences induce painful affects that lead subsequently to repression of associated contents. Indeed, in a previous study in patients undergoing psychodynamic group therapy, we found that negative (painful or aggressive) feelings during confrontation with unresolved conflicts were associated with activation of the ACC as well (Axmacher and Heinemann, 2012). Consistent with our results presented here, these unresolved conflicts were consciously aware to the patients. However, they were often not able to fully accept and tolerate their associated feelings – in other words, these feelings were isolated (a specific defence during which events themselves are not repressed, but associated feelings are; Freud, 1915, p. 153). Although the account of the ACC being involved in conflict monitoring regards information processing in a stricter sense (Botvinick, 2007), it is interesting that the processing of psychodynamic conflicts seems to recruit a similar area.

Our additional question was whether our separation between “apparent conflict” and “absent conflict” reflects a trait (subjects tend to view all sentences as problematic) or rather a state (just the sentences with conflict-related associations cause emotional reactions). Results tend to confirm the conceptualization as a trait. Subjects did rate the sentences with eventual conflict-related associations with more agreement and negative valence. This is in line with the above mentioned assumption that processing of conflicts is rather conscious. The lack of differences in skin conductance (arousal) and brain activity between the two types of sentences speaks against the idea that only the sentences leading to problematic associations have an emotional impact (state). We assume that “apparent conflict” subjects generally tend to show emotional reactions to that type of sentences.

In the vein of this special issue on psychoanalytic neuroscience, the results of our study make a point for the fruitfulness of applying psychoanalytic theory to neuroscientific research. Stimuli were derived from features of typical psychodynamic conflicts and transferred into an fMRI design. It is intriguing that free association, the classical technique most commonly used in psychoanalysis, could be a powerful tool to investigate aspects of conflict processing in neuroimaging, and that the quality of those associations could be used as a genuinely psychodynamic marker to separate subjects into two groups. Other forms of analyses of the associations would have been possible, e.g., qualitative content analysis (Mayring, 1983) or grounded theory (Glaser and Strauss, 1967). Yet, our aim was to stay within a psychoanalytic framework regarding stimulus production and assessment of the associations in order to view the material in a holistic rather than fragmented way. We do think that the general approach of our study could be implemented in other forms of research in neuropsychoanalysis in a fruitful way. Possibilities for future research include the use of individualized stimuli (generated, e.g., through diagnostic OPD-interviews) or recruitment of clinical patients with disorders traditionally believed to result from repressed conflicts (e.g., conversion disorders, or psychogenic, non-epileptic seizures).

### LIMITATIONS

One limitation of the study concerns the sample size. The comparisons between group 1 (“apparent conflict”) and group 2 (“no apparent conflict”) included actually 8 vs. 10 subjects. It is of notice that we obtained significant results with such a small sample size on the group level, but nevertheless, generalizability of results and hence ecological validity remain uncertain. A second limitation lies in the method of the group separation. Raters are experienced psychotherapists trained in psychoanalysis and OPD. They were blind to subjects’ self-reports concerning the sentences and to the process of fMRI analysis. Still, as in any clinical setting, the final decision whether the association to a given sentence actually covers aspects of a psychodynamic conflict of this person remains uncertain.

## Conflict of Interest Statement

The authors declare that the research was conducted in the absence of any commercial or financial relationships that could be construed as a potential conflict of interest.
